# Tools and resources for neuroanatomy education: a systematic review

**DOI:** 10.1186/s12909-018-1210-6

**Published:** 2018-05-03

**Authors:** M. Arantes, J. Arantes, M. A. Ferreira

**Affiliations:** 1 Department of Biomedicine, Unit of Anatomy, Faculty of Medicine of the University of Porto, Al. Prof. Hernâni Monteiro, 4200 – 319, Porto, Portugal; 2 Division of Neuroradiology, Department of Radiology, Portuguese Institute of Oncology, Porto, Portugal; 3 Psychology School, University of Minho, Campus de Gualtar, 4710-057 Braga, Portugal; 4 Department of Public Health, Forensic Sciences and Medical Education, Faculty of Medicine of the University of Porto, Al. Prof. Hernâni Monteiro, 4200 – 319, Porto, Portugal

**Keywords:** Neuroanatomy, Education, Teaching, Learning, Student

## Abstract

**Background:**

The aim of this review was to identify studies exploring neuroanatomy teaching tools and their impact in learning, as a basis towards the implementation of a neuroanatomy program in the context of a curricular reform in medical education.

**Methods:**

Computer-assisted searches were conducted through March 2017 in the PubMed, Web of Science, Medline, Current Contents Connect, KCI and Scielo Citation Index databases. Four sets of keywords were used, combining “neuroanatomy” with “education”, “teaching”, “learning” and “student*”. Studies were reviewed independently by two readers, and data collected were confirmed by a third reader.

**Results:**

Of the 214 studies identified, 29 studies reported data on the impact of using specific neuroanatomy teaching tools. Most of them (83%) were published in the last 8 years and were conducted in the United States of America (65.52%). Regarding the participants, medical students were the most studied sample (37.93%) and the majority of the studies (65.52%) had less than 100 participants. Approximately half of the studies included in this review used digital teaching tools (e.g., 3D computer neuroanatomy models), whereas the remaining used non-digital learning tools (e.g., 3D physical models).

**Conclusions:**

Our work highlight the progressive interest in the study of neuroanatomy teaching tools over the last years, as evidenced from the number of publications and highlight the need to consider new tools, coping with technological development in medical education.

## Background

Among the basic sciences providing relevant medical awareness, human anatomy, which includes gross and neuroanatomy, has historically been considered a key science educational area in medical education [1, 2].

The first descriptions of human anatomy teaching in Europe dates back to Greece, in third century BC, with the introduction of systemic human cadaveric dissection. Although the practice of human dissection was prohibited during the Middle Ages due to religious and popular beliefs, it revival at the beginning of fourteenth century and becomes the core basis in medical education and anatomy teaching until the twentieth century [3, 4]. By that time, significant changes have occurred in undergraduate medical education, on one hand because of the introduction of new subjects into curricular programmes as medical scientific knowledge increases and on the other hand because of the move towards skills-based teaching to face clinical practice [5–8]. Within this new reality, many preclinical medical curricula started to integrate systems-based units, abandoning the traditional, isolated, discipline-based curricular approaches [9–14].

These changing concepts greatly influenced the modern teaching of medical anatomy, with many schools now delivering anatomy using integrated, clinically-oriented modules, with considerably less time allocated to anatomy [15–17]. For example, within the USA contact hours for gross anatomy has fallen from an average of 170 h in 2002 to ~ 150 h in 2012 and in neuroanatomy contact hours decreased from 95 to 83 h from 2002 to 2012 [18]. This general reduction in time dedicated to anatomy teaching at medical schools, associated with the increased demand for clinical importance of the topics covered in anatomy curricula, have led to a redefinition of program content and students’ learning objectives, accompanied by the introduction of innovative teaching and learning approaches. Despite the long history, the role of cadaveric dissection, as the primary tool for anatomical teaching, has been reduced or replaced in most medical schools by prosection, use of plastic models and/or multimedia-based learning packages [19].

Although initially integrated with the teaching of gross anatomy, neuroanatomy can now be found as a stand-alone course or, most frequently, as an integrated part of the systems-based approach, taught alongside other neurosciences. Teaching of neuroanatomy to students is known to be particularly challenging, due to the sheer complexity and interconnectedness of the central nervous system [20]. Students are required to learn not only anatomical structures, but also be able to understand their topography, spatial relationships and clinical significance. In 1994, Jozefowicz [21] introduced the term “neurophobia” as “a fear of the neural sciences and clinical neurology that is due to the students’ inability to apply their knowledge of basic sciences to clinical situations”. In fact, poor teaching and the challenging nature of aspects of neuroanatomy were identified, in one study, as reasons for considering neurosciences/neurology so difficult. To face changes in medical education curricula and to help reduce neurophobia, some anatomists have developed and implemented innovative teaching techniques and strategies. In this context, Moxham et al. [22] also proposed a core syllabus for teaching neuroanatomy to medical students, to provide guidelines concerning neuroanatomical knowledge. However, the debate over how best to teach neuroanatomy in undergraduate medical education continues, with each institution using its own method.

The major aim of the present work is to review the most common methods for teaching neuroanatomy, and their effectiveness. More specifically, we intend to: a) identify the studies that explore neuroanatomy teaching tools; and b) to assess their impact on learning.

## Methods

### Databases searched and search terms

The electronic databases searched in this review included those identified as the most relevant to the topic. More specifically, computer-assisted searches were conducted in six online databases: PubMed, Web of Science, Medline, Current Contents Connect, KCI and Scielo Citation Index. As keywords, four sets were used, combining “neuroanatomy” with “education”, “teaching”, “learning” and “student*”.

### Inclusion and exclusion criteria

The search was restricted to English-language studies that focus on the teaching of neuroanatomy. A comprehensive search was performed for papers available for search from each database’s inception through March 2017. Papers available online ahead of the print version were also analyzed. Manuscripts were included if they were original research studies assessing the impact of using a specific method on student’s learning of neuroanatomy.

The exclusion criteria were as follows: i) descriptive studies on the use of a teaching method without assessing the impact on learning; ii) studies describing the development of a teaching method; iii) studies not focused on the teaching of human neuroanatomy; iv) studies in languages other than English; v) reviews, editorial material, proceeding papers, notes, letters to the Editors and meeting abstracts; and vi) duplicate papers.

### Selection of papers

All databased were reviewed independently by two readers (M.A. and J.A.) using the above stated criteria. More specifically, each manuscript identified was placed on an Excel spreadsheet, and the readers applied the exclusion criteria independently. Disagreements were discussed in a meeting and resolved by consensus. After removal of duplicate manuscripts, all potentially eligible manuscripts were screened by both readers. Then, the full text of all screened manuscripts was carefully read. All data collected was confirmed by a third reader (M.F.), and discussions occurred until a final consensus was reached.

### Charting collating and summarizing the data

Spreadsheets were used to register the most important features of each study, namely the title of the papers, authors, year of publication, university and country where the study was conducted, type and number of participants, teaching tool, aim, methodology, number of participants, and main results/conclusions. Data were summarized, and were then grouped according to these features.

## Results

### Studies included in this review

**Fig. 1:**
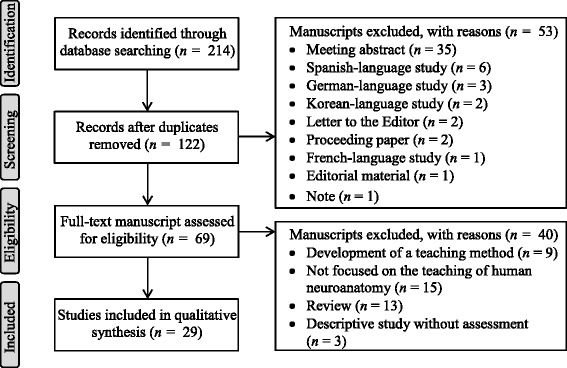
Process applied to identify the manuscripts

The search of PubMed, Web of Science, Medline, Current Contents Connect, KCI and Scielo Citation Index databased yielded 214 manuscripts. After removal of duplicate studies (*n* = 92), a total of 122 manuscripts were identified. On applying the inclusion and exclusion criteria by the two independent readers, 53 manuscripts were excluded because they were written in languages other than English, were abstracts, letters to the Editors, editorial material, proceeding papers, or notes. Therefore, a total of 69 manuscripts were then assessed for eligibility. After these manuscripts were read in their entirety, 40 studies were excluded because they were descriptive reports of a teaching method without assessing its effectiveness, they described the development of a new teaching tool, did not focused on the learning of neuroanatomy (e.g., neuroanatomy of schizophrenic patients) or were review manuscripts. Thus, a total of 29 manuscripts meet the criteria to be included in this review (see Fig. 1).

Table 1 presents some of the features of the papers included in the present work. Of the 29 studies, the first study [36] was published 50 years ago, in 1966. However, the majority of the studies (*n* = 15; 52%) were published in 2012–2016, and 24 (83%) of the studies found were published in the last 8 years. Only 4 (14%) were published before 2005. Most studies were conducted in the United States of America (*n* = 19; 65.52%), followed by the United Kingdom (*n* = 4; 13.79%) and Australia (*n* = 2; 6.90%). The remaining four studies were from Canada, India, Poland and Spain.

**Table 1:** Main features of the manuscripts included in this review (*n* = 29)

Features	Number (%)	Studies
Publication year		
2012–2016	15 (51.72)	I, II, III, IV, V, VI, VII, VIII, IX, X, XI, XII, XIII, XIV, XV
2007–2011	9 (31.03)	XVI, XVII, XVIII, XIX, XX, XXI, XXII, XXIII, XXIV
2002–2007	1 (3.45)	XXV
1966–2001	4 (13.79)	XXVI, XXVII, XXVIII, XXIX
Place of the study		
Australia Canada India Poland Spain UK USA	2 (6.90) 1 (3.45) 1 (3.45) 1 (3.45) 1 (3.45) 4 (13.79) 19 (65.52)	XI, XIII I V XVIII VIII II, III, XII, XXIII IV, VI, VII, IX, X, XIV, XV, XVI, XVII, XIX, XX, XXI, XXII, XXIV, XXV, XXVI, XXVII, XXVIII, XXIX
Type of participants		
Faculty members and students	1 (3.45)	XXII
Master’s degree health care professional students	1 (3.45)	XXI
Non-specified undergraduate students with neuroanatomy experience	4 (13.79)	VI, VIII, XI, XIII
Participants without neuroanatomy experience	3 (10.34)	VII, IX, XVI
Undergraduate or graduate biology students	3 (10.34)	XIV, XVII, XXIV
Undergraduate biomedical students	1 (3.45)	II
Undergraduate medical students	11 (37.93)	I, III, IV, V, XII, XVIII, XX, XXIII, XXVII, XXVIII, XXIX
Undergraduate psychology students	4 (13.79)	X, XIV, XV, XXVI
Undergraduate or graduate physical/ocupational therapy students	3 (10.35)	XIX, XXIV, XXV
Number of participants		
+ 201	3 (10.35)	II, XVIII, XXIII
151–200	3 (10.35)	XIII, XXVII, XXVIII
101–150	4 (13.79)	V, XI, XX, XXIX
51–100	11 (37.93)	IV, VI, VII, VIII, IX, XII, XV, XVI, XIX, XXI, XXII,
0–50	8 (27.59)	I, III, X, XIV, XVII, XXIV, XXV, XXVI
Teaching tool		
Digital tool	14 (50.00)	I, II, IV, VII, VIII, IX, XVI, XIX, XX, XXI, XXII, XXIII, XXV, XXVII
Non-digital tool	14 (50.00)	III, V, VI, X, XI, XII, XIII, XIV, XV, XVII, XXIV, XXVI, XXVIII, XXIX
Type of teaching tool		
3D computer neuroanatomy tools 3D physical models Apps installed in tablets Case studies Computer-based neuroanatomy tools Equivalence-based instruction, EBI Face-to-face teaching Flipped classroom Inquiry-based laboratory instruction Intensive mode of delivery Interpolation of questions Near-peer teaching Renaissance artists’ depictions Self-instructional stations Truncated lectures, conceptual exercises and manipulatives	6 (21.43) 1 (3.57) 1 (3.57) 3 (10.71) 6 (21.43) 2 (7.14) 1 (3.57) 1 (3.57) 1 (3.57) 1 (3.57) 1 (3.57) 1 (3.57) 1 (3.57) 1 (3.57) 1 (3.57)	I, IV, VII, VIII, IX, XVI, XX II XIV XIX, XXI, XXII, XXIII, XXV, XXVII III, XV XIII V VI XI XXIX XII X XXVIII XXIV

Regarding the type of participants, medical students are the most studied sample (*n* = 11; 37.93%), followed by psychology students (*n* = 4; 13.79%), non-specified undergraduate students with neuroanatomy experience (*n* = 4; 13.79%), biology students (*n* = 3; 10.34%), psysical/occupational therapy students (*n* = 3; 10.34%), and volunteers without neuroanatomy experience (*n* = 3; 10.34%). Only one study (3.45%) investigated the effect of a neuroanatomy teaching tool on faculty members. Although 10 studies (34.48%) had 100 or more participants, the remaining 19 (65.52%) had a number of participants less than 100. One study only presented 13 students as participants.

The teaching methods used in the studies included in this review can be classified into digital tools (*n* = 13; 46.43%) and non-digital learning tools (*n* = 15; 53.57%). The digital tools include 3D computer neuroanatomy models, computer-based tools (i.e., computer-aided instruction/learning), and apps installed in tablets. The non-digital tools include the use of case studies, equivalence-based instruction (EBI), 3D physical models, face-to-face teaching, flipped classroom, inquiry-based laboratory instruction, intensive mode of delivery, interpolation of questions, near-peer teaching, Renaissance artists’ depictions, self-instructional stations, and truncated lectures, conceptual exercises and manipulatives.

### Digital tools

#### Computer-based neuroanatomy tools

Table 2 summarizes each study included in this review, including the teaching tool used, aims, methodology employed, and main results/conclusion. Some researchers [23–28] focused their studies on the impact of using computer-based tools for teaching neuroanatomy. More specifically, McKeough et al. [23, 24] investigated the effect of a computer-based tool on students’ performance and their attitudes. Before and after test questions revealed that scores improved significantly after working with the learning model. In addition, students reported the computer-aided neuroanatomy learning modules as a valuable and enjoyable learning tool, and perceived their clinical-self efficacy as higher as a result of working with them.

**Table 2:** Summary description of the 29 studies included in this review (listed by year of publication)

Title	Authors (year)	University, country	Participants (number)	Teaching tool	Aim	Methodology	Main results/conclusion
Evaluation of an online three-dimensional interactive resource for undergraduate neuroanatomy education^I^	Allen et al. (2016)	Western University, Canada	Second-year undergraduate medical students (*n* = 47)	3D computer neuroanatomy model (*digital tool*)	To evaluate the impact on learning of a novel, interactive 3D neuroanatomy model.	Participants were divided into 2 groups, each accessing 2 learning modalities (3D model and a cadaveric laboratory session). After each modality, students completed a test. (*mix between- and within- subject design*)	Participants were pleased to work with the 3D model. In addition, their learning outcomes significantly improved after accessing this teaching tool.
Mobile technology: students perceived benefits of apps for learning neuroanatomy^II^	Morris et al. (2016)	University of Leeds, UK	Undergraduate biomedical students enrolled in a level 2 course (*n* = 519)	5 apps installed in tablet (Apple iPad) devices (*digital tool*)	To examine the students’ use of apps in a neuroanatomy practical class: their perceptions and learning outcomes.	There were three cohorts of students (3-year study). The neuroanatomy practical session included the use of tablet devices. After the session the students completed a questionnaire about the use of the apps.	Students made extensive use of the apps, considered them easy to use and beneficial for learning. Compared with the year before the trial started, there was an increase in the students’ performance.
The student experience of applied equivalence-based instruction for neuroanatomy teaching^III^	Greville et al. (2016)	Swansea University, UK	First- and second-year undergraduate medical students (*n* = 43)	Equivalence-based instruction, EBI (*non-digital tool*)	To develop and assess the effectiveness of EBI learning resources.	Initially participants completed a pre-test. Then the learning of the relations occurred, followed by a post-test design to assess students’ learning. (*within-subject design*)	The EBI resources were an effective, efficient and well-received method for teaching neuroanatomy.
Development and assessment of a new 3D neuroanatomy teaching tool for MRI training^IV^	Drapkin et al. (2015)	Brown University, USA	First-year undergraduate medical students (*n* = 73)	3D computer neuroanatomy model (*digital tool*)	To create and evaluate the efficacy of a computerized 3D neuroanatomy teaching tool	Participants were divided into two groups. The first group was taught using the 3D teaching tool, and the second using traditional methods. Scores from an MRI identification quiz and survey were compared. (*between-subject design*)	The 3D teaching tool was an effective way to train students to read an MRI of the brain and particularly effective for teaching C-shaped internal brain structures.
Perception of MBBS students to “flipped class room” approach in neuroanatomy module^V^	Veeramani et al. (2015)	Jawaharlal Institute of Postgraduate Medical Education and Research, India	First-year undergraduate medical students (*n* = 130)	Flipped classroom (*non-digital tool*)	To examine the impact of a flipped classroom approach (i.e., assess students’ perception and the impact on their performance and attitudes)	Pre- and post-tests were designed to test the learning aims of the session. The perception of the students was also assessed. (*within- subject design*)	Results showed significant differences between the pre and post-test scores. Student response to the flipped classroom structure was largely positive
A mind of their own: Using inquiry-based teaching to build critical thinking skills and intellectual engagement in an undergraduate neuroanatomy course^VI^	Greenwald & Quitadamo (2014)	A regional university in the Pacific Northwest, USA	Undergraduate students enrolled in a human neuroanatomy course (*n* = 85)	Inquiry-based clinical case, IBCC (*non-digital tool*)	To determine which teaching method (conventional and IBCC) produces greater gains in critical thinking and content knowledge	Participants were divided into two groups: conventional and Experimental group. All students were analyzed for exam and course grade performance. Critical thinking pre- and post- tests were analyzed. (*mix between- and within- subject design*)	Using the California critical thinking skills test, students in the conventional neuroanatomy course gained less than 3 national percentile ranks, whereas IBCC students gained over 7.5 within one academic term.
Computer-based learning: graphical integration of whole and sectional neuroanatomy improves long-term retention^VII^	Naaz et al. (2014)	University of Louisville, USA	Volunteers between 16 and 34 years, with minimal or no knowledge of neuroanatomy (*n* = 64)	3D computer neuroanatomy models (*digital tool*)	To examine if instruction with graphically integrated representations of whole and sectional neuroanatomy is effective	Participants were divided into two groups. After a pretest, participants learned sectional anatomy using one of the two learning programs: sections only or 2-D/3-D. Then tests of generalization were completed. (*between- subject design*)	It showed that the use of graphical representation helps students to achieve a deeper understanding of complex spatial relations
Enhancing neuroanatomy education using computer-based instructional material^VIII^	Palomera et al. (2014)	University of Salamanca, Spain	Undergraduate students enrolled in a medical anatomy course (*n* = 65)	3D computer neuroanatomy models (*digital tool*)	To develop a computer-based tool based on 3D images, and to examine if the for this tool depends on their visuospatial ability	Participants completed an online rating-scale to measure the educational value that they assigned to the teaching tool. In addition, the student’s visuospatial aptitude was assessed. (*between-subject design*)	Findings showed that students assigned a high educational value to this tool, regardless of their visuospatial skills.
Computer-based learning: Interleaving whole and sectional representation of neuroanatomy^IX^	Pani et al. (2013)	University of Louisville, USA	Undergraduate students with rudimentary knowledge of neuroanatomy (*n* = 59)	3D computer neuroanatomy model (*digital tool*)	To compare a basic transfer method for learning whole and sectional neuroanatomy with a method in which both forms of representation were interleaved.	There were 3 experimental conditions: i) section only, ii) whole then sections; and iii) alternation. (*between-subject design*)	Interleaved learning of whole and sectional neuroanatomy was more efficient than the basic transfer method.
Da Vinci coding? Using renaissance artists’ depictions of the brain to engage student interest in neuroanatomy^X^	Watson (2013)	Lewis & Clark College, USA	Undergraduate psychology students (*n* = 27)	Renaissance artists’ depictions of the central nervous system (*non-digital tool*)	To increase students’ interest in the study of neuroanatomy	There were interactive classroom exercises using well-known Renaissance artists’ depictions of the brain. Then participants completed a feedback questionnaire.	These exercises increased the interest of the students in the topic. The authors suggest that these exercises may be a useful addition to courses that introduce or review neuroanatomical concepts.
Intensive mode delivery of a neuroanatomy unit: Lower final grades but higher student satisfaction^XI^	Whillier, & Lystad, (2013)	Macquarie University, Australia	Undergraduate students enrolled in the traditional and intensive neuroanatomy units (*n* = 125)	Intensive mode of delivery (*non-digital tool*)	To compare the intensive and traditional units of neuroanatomy for undergraduate students.	The intensive mode neuroanatomy unit showed the students the same quantity and quality of material to the same standard, including the hours. However, the material was delivered in a shorter timeframe. (*between-subject design*)	Students obtained lower final grades in the new intensive mode delivery but reported having similar overall satisfaction with their laboratory practical classes.
Near-peer teaching in clinical neuroanatomy^XII^	Hall et al. (2013)	University of Southampton, UK	Undergraduate medical students (*n* = 60)	Near-peer teaching (*non-digital tool*)	To develop and deliver a near-peer programme of study.	Two medical students organized and delivered the teaching to their colleges in a series of seven sessions. At the end of each session, participants were asked to fill a feedback questionnaire. (*within-subject design*)	Students’ perceived level of knowledge increased after the near-peer teaching sessions, supporting the use of this teaching tool in neuroanatomy courses.
The effect of face-to-face teaching on student knowledge and satisfaction in an undergraduate neuroanatomy course^XIII^	Whillier & Lystad, (2013)	Macquarie University, Australia	Undergraduate students enrolled in the new and old neuroanatomy units (*n* = 181)	Face-to-face teaching (*non-digital tool*)	To examine if face-to-face teaching has an impact on student performance and overall satisfaction with the course.	Two groups of students (old and restructured unit of neuroanatomy) were analyzed. A questionnaire was used to compare them in terms of the rate they gave to the course, satisfaction, and final grades. (*between-subject design*)	The increase in total face-to-face teaching hours in the restructured unit of neuroanatomy does not improve student grades. However, it does increase student satisfaction.
Using case studies as a semester-long tool to teach neuroanatomy and structure-function relationships to undergraduates^XIV^	Kennedy (2013)	Denison Univer, USA	Undergraduate biology and psychology students (*n* = 50)	Case studies (*non-digital tool*)	To investigate the effect of teaching neuroanatomy through presentation and discussion of case studies.	Students were expected to collaborate with their colleges in small groups. In each case study, the entire class was asked to participate in some aspect of the case.	Students report enjoying learning brain structure using this method, and commented positively on the class activities associated with learning brain anatomy.
Using equivalence-based instruction to increase efficiency in teaching neuroanatomy^XV^	Pytte & Fienup (2012)	Queens College, USA	Undergraduate students, primarily of psychology majors (*n* = 93)	Equivalence-Based Instruction, EBI (*non-digital tool*)	To study if EBI is effective in teaching neuroanatomy in a large classroom setting	Fourteen brain regions were identified, and conditional relations were explicitly taught during three lectures. Then, students completed test to evaluate some relations that were taught and some not taught.	Selection of associations by the teacher can encourage the spontaneous emergence of novel associations within a concept or category. Therefore, it can increase the efficiency of teaching.
Computer-based learning of neuroanatomy: a longitudinal study of llearning, transfer, and retention^XVI^	Chariker et al. (2011)	University of Louisville, USA	Undergraduate students who reported minimal knowledge of neuroanatomy (*n* = 72)	3D computer neuroanatomy model (*digital tool*)	To determine the effectiveness of new methods for teaching neuroanatomy with computer-based instruction	Using a 3D graphical model of the human brain, students learned either sectional anatomy alone (with perceptually continuous or discrete navigation) or whole anatomy followed by sectional anatomy. Participants were tested immediately and after 2–3 weeks. (*between-subject design*)	Results showed efficient learning, good long-term retention, and successful transfer to the interpretation of biomedical images. They suggest that computer-based learning can be a valuable teaching tool.
Human brains engaged in rat brains: student-driven neuroanatomy research in an introductory biology lab course^XVII^	Gardner et al. (2011)	Purdue University, USA	First-year undergraduate students interested in majoring in biology (*n* = 13)	Inquiry-based laboratory instruction (*non-digital tool*)	To increase student interest in biology by exposing them to novel research projects	Students acquired basic lab skills within the context of a research question of a member of the faculty. Biology students who were not taking the research-based, Bio-CASPiE course, formed the comparison group for pre- and post-semester questionnaires. (*mix between-within- subject design*)	The inquiry-based laboratory instruction increased students’ motivation and excitement, encouraged good scientific practices, and can potentially benefit departmental research.
The study techniques of Asian, American, and European medical students during gross anatomy and neuroanatomy courses in Poland^XVIII^	Zurada et al. (2011)	Medical University in Poland, Poland	International medical students, from the Polish, American, and Taiwanese divisions (*n* = 705)	–	To investigate similarities and differences among American, Asian, and European medical students in terms of their study methods.	Participants completed a questionnaire in which they reported which methods they used to study, and which of the methods they believed were most efficient for comprehension, memorization, and review. (*between-subject design*)	Results showed some differences in study techniques among students from the different ethnic back- grounds (e.g., Polish and American preferred the use of dissections and prosected specimens)
Effectiveness of a computer-aided neuroanatomy program for entry-level physical therapy students: anatomy and clinical examination of the dorsal column-medial lemniscal system^XIX^	McKeoughet al. (2010)	California State University & University of the Pacific, USA	Undergraduate physical therapy students (*n* = 61)	Computer-aided instruction, CAI (*digital tool*)	To determine if a computer- aided instruction learning module improves students’ neuroanatomy knowledge	Students completed a paper-and-pencil test on the neuroanatomy/physiology and clinical examination of the DCML system, both before and after working with the teaching tool (*within-subject design*)	Findings showed that clinical examination post-test scores improved significantly from the pre-test scores
A Novel Three-Dimensional Tool for Teaching Human Neuroanatomy^XX^	Estevez et al. (2010)	Boston University School of Medicine, USA	First-year undergraduate medical students (*n* = 101)	3D physical neuroanatomy model (*digital tool*)	To develop and assess a new tool forg teaching 3D neuroanatomy to first-year medical students	First, all students were presented to traditional 2D methods. Then, the experimental group constructed 3D color-coded physical models, while the control group re-examined 2D brain cross-sections. (*between-subject design*)	3D computer model was an effective method for teaching spatial relationships of brain anatomy and seems to better prepare students for visualization of 3D neuroanatomy
Attitudes of health care students about computer-aided neuroanatomy instruction^XXI^	McKeough& Bagatell (2009)	A University in the West Coast, USA	Master’s degree health care professional students (*n* = 77)	Computer-aided instruction, CAI (*digital tool*)	To examine students’ attitudes toward CAI, which factors help their development, and their implications.	Three computer-aided neuroanatomy learning modules were used. Students independently reviewed the modules as supplements to lecture and completed a survey to evaluate teaching effectiveness (*within-subject design*)	The CAI modules examined in this study were effective as adjuncts to lecture in helping the students learn and make clinical applications of neuroanatomy information
A usability study of users’ perceptions toward a multimedia computer-assisted learning tool for neuroanatomy^XXII^	Gould et al. (2008)	University of Kentucky & University of Louisville & Nova Southeastern University, USA	Faculty and students from some institutions across the country (*n* = 62)	Computer-aided instruction, CAI (*digital tool*)	To assess users’ perceptions of the computer-based tool: “Anatomy of the Central Nervous System: A Multimedia Course”	First, participants used the multimedia prototype. Then they completed a usability questionnaire designed to measure two usability properties: program need and program applicability	This study showed that the CAI was well-designed for all users, and demonstrates the importance of integrating quality properties of usability with principles of human learning.
Attitudes to e-learning, learning style and achievement in learning neuroanatomy by medical students^XXIII^	Svirko & Mellanby (2008)	Oxford University, UK	Second-year pre-clinical undergraduate medical students (*n* = 205)	Computer-aided learning, CAL (*digital tool*)	To investigate the impact of an online course on encouraging students to learn and their academic performance.	The students approach to learning towards the CAL course and towards their studies in general was compared. Student attitudes and ratings were also assessed.	Findings revealed that students reported using significantly less deep approach to learning for the CAL course.
Using truncated lectures, conceptual exercises, and manipulatives to improve learning in the neuroanatomy classroom^XXIV^	Krontiris-Litowitz (2008)	Youngstown State University, USA	Undergraduate biology and graduate biology and physical therapy students (*n* = 19)	Truncated lectures, conceptual exercises, and manipulatives (*non-digital tool*)	To use truncated lectures, conceptual exercises, and manipulatives to make learning more effective and increase critical thinking	The curriculum was revised. More specifically, it became shorter, included practice problems that presented the spinal tracts in an applied context, and included a manipulative. Student’s learning was then assessed and compared with previous classes. (*between-subject design*).	Students’ learning was more effective under the revised curriculum, suggesting that this revised curriculum could potentially be applied to other topics.
Design and utility of a web-based computer-assisted instructional tool for neuroanatomy self-study and review for physical and occupational therapy graduate students^XXIV^	Foreman et al. (2005)	University of Utah, USA	Undergraduate physical therapy and occupational therapy students (*n* = 43)	Computer-assisted instruction, CAI (*digital tool*)	To develop a CAI, and assess their design and utility to teach neuroanatomy.	A questionnaire addressed navigation, clarity of the images, benefit of the CAI tool, and students rating. Students were also asked to compare this tool with traditional learning tools.	Design and utility of a web-based computer-assisted instructional tool for neuroanatomy self-study and review for physical and occupational therapy graduate students^XXIV^
A neuroanatomy teaching activity using case studies and collaboration^XXVI^	Sheldon (2000)	University of Michigan, USA	Undergraduatestudents of an introductory psychology course (*n* = 28)	Case studies and collaboration (*non-digital tool*)	To evaluate an easier and less time-consuming method for teaching neuroanatomy.	Students collaborated and applied their neuroanatomy knowledge to several case studies during classes.	Findings showed that students assessed this method as very enjoyable and helpful for remembering or learning the material.
Computer-based neuroanatomy laboratory for medical students^XXVII^	Lamperti & Sodicoff (1997)	Temple University, USA	First-year undergraduate medical students (*n* = 185)	Computer-based neuroanatomy laboratory (*digital tool*)	To develop a computer-based laboratory program to substitute the traditional glass-slide laboratory	They compared the performances of those classes that previously had the traditional laboratory with two succeeding classes that used computer program (*between-subject design*).	Test scores showed that the students’ performance on laboratory material was similar in both classes.
Teaching of neuroanatomy by means of self-instructional laboratory stations^XXVIII^	Fisher et al. (1980)	University of Michigan Medical School, USA	First-year undergraduate medical students (*n* = 200)	Self-instructional stations (*non-digital tool*)	To teach neuroanatomy using self-instructional laboratory stations	Neuroanatomical laboratory material was presented in a series of six self-instructional stations. Five weeks later, short examinations tests occurred.	Results of the tests indicated a mastery of station material as defined by the objectives and an ability to use the material in applied problems.
Cognitive processes in learning neuroanatomy^XXIX^	Geeartsma & Matzke (1966)	University of Kansas, USA	First-year undergraduate medical students (*n* = 107)	Interpolation of questions (*non-digital tool*)	To investigate the effect of interpolation of questions into a lecture presentation.	Participants were divided into 2 matched groups. Each group was shown lectures on the visual system which differed only in regard to taxomic level of 10 questions posed lecture. Performance was then measured (*between-subject design*).	Emphasis on recall questions aids performance on subsequent recall questions more than emphasis on problem-solving questions helps in the solution of subsequent problem-solving questions.

Foreman et al. [25] conducted a prospective evaluation by asking questions to the students regarding tool navigation and benefits, clarity of the images, and by requesting them to compare these tools to traditional ones. Results showed that most of the students agreed that the computer-based tool was easy to navigate and overall beneficial, educational in structure identification and had clear images, and somewhat better than traditional learning tools.

Only one study [26] assessed the perception of both students and faculty members with a computer-based tool. Analysis of the results supported the research hypotheses that the prototype was well-designed for different types of users in various educational contexts, and that would be useful as a neuroanatomy review tool for health-professions students.

The studies mentioned above suggested that computer-based tools seem to be effective in teaching neuroanatomy. Lamperti and Sodicoff [27] compared students’ performance between those classes that previously had the traditional anatomy laboratory with two succeeding classes that used the computer-based laboratory. When assessing the total performances, results showed no statistically significant differences in the average grades for classes during the 2 years prior to and 2 years following the introduction of computers in the course. However, Svirko and Mellanby [28] compared the students’ approach to learning for a computer-based course in Neuroanatomy with that for their studies in general. Students reported lower deep approach scores (seeking the meaning of the information being taught) and higher surface approach scores (rote-learning motivated by fear of failure and without integrating current and previous knowledge) for the computer-aided course than for their studies in general. Also, only approximately one quarter of the students agreed or strongly agreed they enjoyed this course.

#### 3D computer neuroanatomy tools

More recently, studies have used three-dimensional (3D) computer graphical models of human brain as a teaching tool in neuroanatomy classes [29–34]. For example, Drapkin et al. [29] compared students’ performance when learning through a new 3D program or through traditional methods. They divided the students into two groups: an experimental (3D program) and a control group (traditional). Results showed that scores extracted from questions involving C-shaped internal brain structures were higher for the experimental group, and that these students reported higher confidence levels. Allen et al. [30] also divided the students into two groups, but each group was exposed to two types of teaching resources, presented in a contrabalanced order: 3D new learning module and cadaveric laboratory session. After accessing each teaching resource, participants completed a test. Findings showed that participants who initially learned using the 3D learning module scored significantly better than students who learned using the gross anatomy resources. In addition, scores significantly improved for students who accessed the 3D learning module following exposure to the cadaveric resources. Palomera et al. [31] assessed if students’ evaluation of a new 3D computer-based tool depended on their visuospatial skills to establish handle spatial relationships. Findings revealed that students with both high visuospatial ability and low visuospatial ability assigned similar high educational value to this tool.

Naaz et al. [32], Pani et al. [33] and Chariker et al. [34] focused on the learning of whole and sectional neuroanatomy using neuroanatomical 3D computer models. Their findings suggested that: i) explicit graphical demonstration of the spatial relations between 3D whole anatomy and 2D sectional anatomy leads to high long-term retention of sectional neuroanatomy [32]; ii) an integrative learning method, that presents whole and sectional neuroanatomy in alternating trials, increases the students’ performance [33]; and iii) instruction of neuroanatomy designed on the basis of substantial transfer of learning from whole to sectional anatomy is an effective method for teaching neuroanatomical structures [34].

#### Other digital tools

Only one study used tablet devices (Apple iPads) in neuroanatomy practical sessions to investigate the effectiveness of specific apps in students’ perceptions and performance [35]. Results showed that the students considered the apps to be beneficial for learning. In addition, their performance in neuroanatomy-related questions increased after the introduction of the tablet devices.

#### Non-digital tools

Regarding the use of non-digital tools, there is a variety of resources that can be used in neuroanatomy classes. In their pioneer work, published in 1966, Geeartsma and Matzke [36] investigated the effect of interpolation of questions into a lecture presentation. Findings revealed that the emphasis on recall questions led to an increase in students’ performance on subsequent recall test questions.

Krontiris-Litowitz [37] studied a revised curriculum using truncated lectures, conceptual exercises, manipulatives, and that was shorter. She found that students’ learning was more effective under this new curriculum. Whillier & Lystad [38] also compared units of neuroanatomy for undergraduate students: an intensive mode and a traditional mode. Even though students showed similar levels of satisfaction, grades were lower in the new intense mode. In addition, Whillier & Lystad [39] compared two other units of neuroanatomy – an old and a restructured unit. Results showed that the increase in total face-to-face teaching hours in the restructured unit led to an increase in the students’ satisfaction. However, it does not improve their grades.

Sheldon [40], Kennedy [41] and Greenwald and Quitadamo [42] included several case studies during classes and students were expected to collaborate and participate in their discussions. Results showed that students evaluated this method as enjoyable, helpful for remembering or learning the material, commented positively on the class activities and gained more national percentile ranks than students in a conventional neuroanatomy course. Watson [43] used interactive classroom exercises using well-known Renaissance artists’ depictions of the brain, and found that these exercises increase the interest of the students in neuroanatomy. Veeramani et al. [44] investigated the impact of another method that also aims to increase students’ collaboration and participation in class: a flipped classroom approach. In this method, students are expected to attend the class with basic understanding of the subject to be able to participate and engage in discussions. Findings revealed that most of the participants felt that the work-sheet with questions provided before class allowed them to adquired a deeper understanding of the subject and believed that the resources provided increased their interest to read.

Fisher et al. [45] used neuroanatomy self-instruction laboratory stations to present neuroanatomical laboratory material making the students’ more active in their learning process. Test results indicated a mastery of station material and positive students’ attitudes. Gardner et al. [46] exposed students to novel research projets into their laboratory experience. Findings showed that working within the context of a research question of a member of the faculty increase students’ motivation and excitement, and encouraged good scientific practice. Hall et al. [47] developed and delivered a near-peer programme of study, in which two medical students delivered the teaching to their colleges, aiming them to grow through their similar knowledge base and shared experiences. After a series of seven sessions, students perceived their level of knowledge as being higher.

Pytte and Fienup [48] and Greville et al. [49] used equivalence-based instruction (EBI) as a tool for teaching neuroanatomy to undergraduate students – teaching how physically disparate stimuli are functionally equivalent, or interchangeable. Findings suggested that: i) selection of associations by the teacher can led to the spontaneous emergences of novel associations within a concept or category; and ii) EBI is a useful tool to teach students to read an MRI of the brain and speciflly useful for teaching C-shaped internal brain structures.

Finaly, even though the use of 3D computer models to teach neuroanatomy has been increasing since 2011, 1 year before Estevez et al. [50] developed and assessed a 3D physical tool. Whereas the control group was exposed to 2D brain cross-sections, the experimental group constructed 3D color-coded physical models. Test results showed that the overall quiz scores for the experimental group were significantly higher than the control group. However, only the scores for questions requiring 3D visualization were significantly higher in the experimental group.

#### Cross-cultural comparisons

Zurada et al. [51] investigated similarities and differences in study methods among American, Asian, and European medical students. For that, the authors asked participants to fill a questionnaire reporting the study methods they use to study, and which methods they believed were the most effective in terms of comprehension, memorization, and review. Results revealed differences in the study techniques among students from the different countries. For example, Polish and American tended to prefer the use of dissection and prosected specimens compared to the Taiwanese students.

## Discussion

From searching PubMed, Web of Science, Medline, Current Contents Connect, KCI and Scielo Citation Index databases, 29 studies were identified. Even though the first study was published 50 years ago, more than four-fifths of the studies were published in the last 8 years, evidencing a growing awareness of this thematic over the most recent years. In fact, several modifications in anatomy and, in particular, in neuroanatomy education have been made over the last few decades, and numerous strategies have been recognized to increase the performance of students [52]. The studies emerged from different countries, including United States of America, United Kingdom, Australia, Canada, India, Poland and Spain. This indicates that the interest for the teaching of neuroanatomy is cross-cultural. However, the paper published by Zurada et al. [51] showed that there are some differences in the study methods adopted by medical students from different countries, such as American, Asian, and European.

Concerning the type of participants, medical students are the most studied sample, which is not surprising taking in consideration that, although several changes have occurred in medical curricula worldwide, the anatomic background is still considered a keystone for approaching clinical medicine [53]. Regarding the number of participants, approximately two-thirds used a sample of 100 participants or less, leading to a low average statistical power. This is a concern regarding published literature in this area, as many of the studies found in this review have limitations imposed by sample size. For example, Gardner et al. [46] investigated only 13 students, Krontiris-Litowitz [37] 19 students, Watson [43] 27, and Sheldon [40] 28 students. A small statistical power is known to reduce chance of detecting a true effect, as well as to reduce the likelihood that a statistically significant result reflects a true effect [54].

In terms of teaching methods used in the studies included in this review, almost half of them used digital tools, such as computer-based tools, 3D computer neuroanatomy models and apps installed in tablets. The majority of the six studies that focused on computer-based neuroanatomy tools showed that: i) it is well-designed for both students and faculty members; ii) the performance of the students increased after working with the learning model; and iii) the students had positive attitudes towards these tools. However, the two remaining studies found that there were no statistically significant differences in the average grades of the students after the introduction of computers in the course, and that students reported lower deep approach scores for the computer-aided course than for their studies in general. These results suggest that even though the computer-based tools seem to be effective in teaching neuroanatomy in certain contexts, this assumption cannot be generalized without further research.

Since 2014, half of the studies focused on 3D computer-based tools, highlighting a growing interest in exploring 3D models on learning of neuroanatomy. Overall, these studies revealed that this digital tool is an effective method for teaching neuroanatomical structures. Findings also showed that students assigned a high educational value to this tool. These results are somewhat inconsistent with those from Azer and Azer [55] who concluded in their review that there is no evidence that the use of 3D models is superior to traditional tools for teaching anatomy. It is possible that the structure of the brain have some particularities that require more the use of the students’ visual-spatial abilities than other anatomical structures of the body. Therefore, the use of 3D tools, by facilitating the mental rotation and manipulation of the brain structures, may facilitate the learning of neuroanatomy.

The non-digital tools include a variety of resources used in neuroanatomy classes.

Findings revealed that the following strategies led to an increase in students’ performance and positive attitudes: i) emphasis on recall questions; ii) use of case studies; iii) inclusion during class of truncated lectures, conceptual exercises, and manipulatives; iv) practice of exercises using well-known Renaissance artists’ depictions of the brain; v) adoption of the flipped classroom approach; vi) use of neuroanatomy self-instruction laboratory stations; vii) inclusion of novel research projets into the laboratory experience; viii) near-peer programmes; ix) EBI; and x) 3D physical models. The increase in total face-to-face teaching opportunities was shown to increase students’ satisfaction but not their grades, and teaching neuroanatomy in an intense mode was shown to lower students’ grades compared to a traditional mode.

### Limitations

Even though a rigorous approach was adopted to undergo this systematic literature review, our study presents some limitations. First, we restricted our search to six databases: PubMed, Web of Science, Medline, Current Contents Connect, KCI and Scielo Citation Index databases. Thus, it is possible that some studies addressing our aim could be found if searches in other databases were conducted. Second, in our search, four sets of keywords were used, combining “neuroanatomy” with “education”, “teaching”, “learning” and “student*”. It is also possible that some studies may focus on neuroanatomy teaching tools but use other terminology to describe them. Third, this review included only papers written in English, and therefore 12 out of 117 studies were eliminated. Some of those papers written in languages other than English may address the aim of our study, and we did not consider them. Fourth, even though all studies were carefully reviewed independently by two readers, and all data collected was confirmed by a third reader, data may been biased by the subjectivity of the readers.

## Conclusions

Our work highlights the progressive interest in the study of neuroanatomy teaching tools over the last 8 years, as evidenced from the number of publications. The view of the different strategies to teach neuroanatomy, may provide guidelines for curricular improvements in this complex area of medical education.

## Abbreviations

3D: Three-dimensional
CAI: Computer-aided instruction
CAL: Computer-aided learning
DCML: Dorsal column-medial lemniscal
EBI: Equivalence-based instruction
IBCC: Inquiry-based clinical case
